# Supplementary material for: Prediction of hyperbolic exciton-polaritons in monolayer black phosphorus
Source: Nat Commun. 2021 Sep 24;12:5628. doi: 10.1038/s41467-021-25941-5 (PMC8463555; doi:10.1038/s41467-021-25941-5)
Supplement: Supplementary file 1 — Supporting Information [file 41467_2021_25941_MOESM1_ESM.pdf]

Supplementary Information

Prediction of hyperbolic exciton-polaritons in monolayer black phosphorus

Fanjie Wang et al.

Supplementary Note I: Extraction of the optical conductivity

In this note, we will extract the optical conductivity of monolayer black phosphorus (BP), few-layer BP, and monolayer transition-metal-dichalcogenides (TMDCs) from experimental spectra.

The complex sheet optical conductivity (σ) of monolayer BP can be determined from the reflection contrast spectrum through the following expression[1]:

$$\frac{R}{R_0} - 1 = \frac{\left|\frac{1-n_s-Z_0\sigma}{1+n_s+Z_0\sigma}\right|^2}{\left|\frac{1-n_s}{1+n_s}\right|^2} - 1, \quad (1)$$

where Z_0 is the vacuum impedance, n_s is the refractive index of the substrate ($n_s = 1.39$ for the PDMS substrate). For few-layer BP (layer number >1), the infrared extinction spectra were obtained in the spectral range from 0.36 eV to 1.36 eV. The relationship between the sheet optical conductivity σ and the extinction spectra reads[1]:

$$1 - \frac{T}{T_0} = 1 - \left|\frac{1+n_s}{1+n_s+Z_0\sigma}\right|^2, \quad (2)$$

Generally, the sheet optical conductivity σ, excluding Drude response of possible free carriers, can be modeled by a superposition of Lorentzian oscillators:

$$\sigma = \frac{i}{\pi}\sum_{k=1}^{N} \frac{\omega S_k}{\omega^2 - {\omega_k}^2 + i\omega\gamma_k}, \quad (3)$$

Here, S_k is the spectral weight, ω_k is the inter-band transition frequency, and γ_k is the resonance width of the k_{th} transition resonance. However, to ensure the validity of fitting results, we rewrite the Supplementary Equation (3):

$$\sigma = -id\omega\varepsilon_0\chi = -id\omega\varepsilon_0(\varepsilon - 1), \quad (4)$$

where d is the sheet thickness ($d = 0.53$ nm for monolayer BP), ε_0 is the vacuum permittivity. For 2L-5L BP, the thickness d is assumed to vary linearly with layer number (*M*): $d = 0.53M$ nm. Moreover,

$$\varepsilon(\omega) = 1 - \sum_{k=1}^{N} \frac{f_k}{\omega^2 - {\omega_k}^2 + i\omega\gamma_k}, \quad (5)$$

is the permittivity, where f_k denotes the oscillator strength. In the limit of $\omega \to 0$,

$$\varepsilon(0) = 1 + \sum_{k=1}^{N} \frac{f_k}{{\omega_k}^2}, \quad (6)$$

giving the static dielectric constant.

Combining Supplementary Equation (5), Equation (4), and Equation (1), we fitted the experimental spectra of the monolayer BP along the armchair direction. The fitting parameters (oscillator strength f_k, peak energy ω_k and linewidth γ_k of each oscillator) in Supplementary Equation (5) were then determined from the fitting. With all of the parameters in hand, we obtained the dielectric function ε according to Supplementary Equation (5). The optical conductivity σ was eventually determined by substituting Supplementary Equation (5) into Supplementary Equation (4). Given the asymmetric line-shape of the 1*s* resonance state[2, 3], multi-oscillators were utilized, which gives an almost perfect fitting result, as shown in Supplementary Fig. 1a. The color curves there indicate the contributions from each oscillator. The corresponding fitted dielectric function ε is presented in Supplementary Fig. 1b. In our analysis, at the zero frequency ($\omega \to 0$), the fitting results yield a $\varepsilon(0) = 12$, which agrees well

with the theoretical[4, 5] and experimental values determined by the far-infrared interference spectra[6]. This validates our fitting results.

For the polarization along the zigzag direction, we rewrite the $\varepsilon(\omega)$:

$$\varepsilon(\omega) = \varepsilon_{\mathrm{b}} - \sum_{k=1}^{N} \frac{f_k}{\omega^2 - {\omega_k}^2 + i\omega\gamma_k} \quad (7)$$

The contributions from higher frequency oscillators beyond our measurement range are all accounted by the background dielectric constant ε_{b}. Keeping in mind of the anisotropy of the BP, for the response along the zigzag direction, we adjusted the parameter ε_{b} ($\varepsilon_{\mathrm{b}} = 3.9$) to achieve $\varepsilon(0) = 10$ (ref. 7). Using Supplementary Equation (7), Equation (4), and Equation (2), we extracted the parameters of oscillators (f_k, ω_k and γ_k) by fitting the experimental spectra. The fitted result of monolayer BP along the zigzag direction is shown in the inset of Supplementary Fig. 1a, without prominent transition peaks.

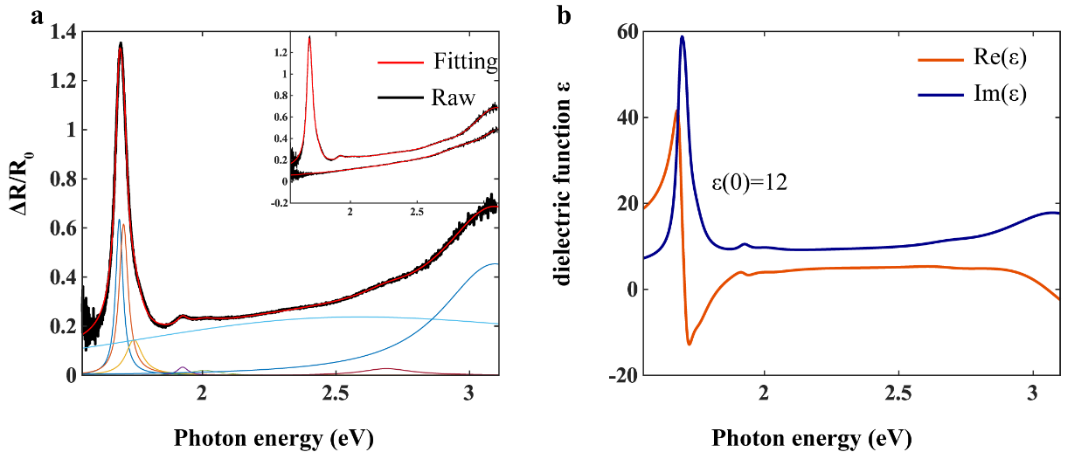

Supplementary Figure 1 | Fitting result of 1L BP. a Reflection spectrum of 1L BP (black curve) and the fit (red curve) along the armchair direction. The color curves represent the contributions of its individual components. The inset shows the overall fitting results along two crystallographic axes, armchair and zigzag directions, where the fits (red curves) almost perfectly overlap the experimental data. **b** Fitted dielectric function along the armchair direction. The fitting result yields a static dielectric constant $\varepsilon(0) = 12$ along the armchair direction.

For extinction spectra of 2L-5L BP along the armchair direction, using Supplementary Equation (7), Equation (4), and Equation (2), by adjusting the parameter ε_b to achieve the monolayer value $\varepsilon(0) = 12$, we got good fitting results, as shown in the Supplementary Fig. 2. The fitted parameters (f_k, ω_k and γ_k) of each oscillator were then used to plot the optical conductivity σ and dielectric function ε, as shown in Supplementary Figs. 2e-i. The pink areas in Supplementary Fig. 2e and Fig. 2f indicate $\mathrm{Im}(\sigma_{AC}) > 0$ regimes, where hyperbolic exciton-polariton modes exist.

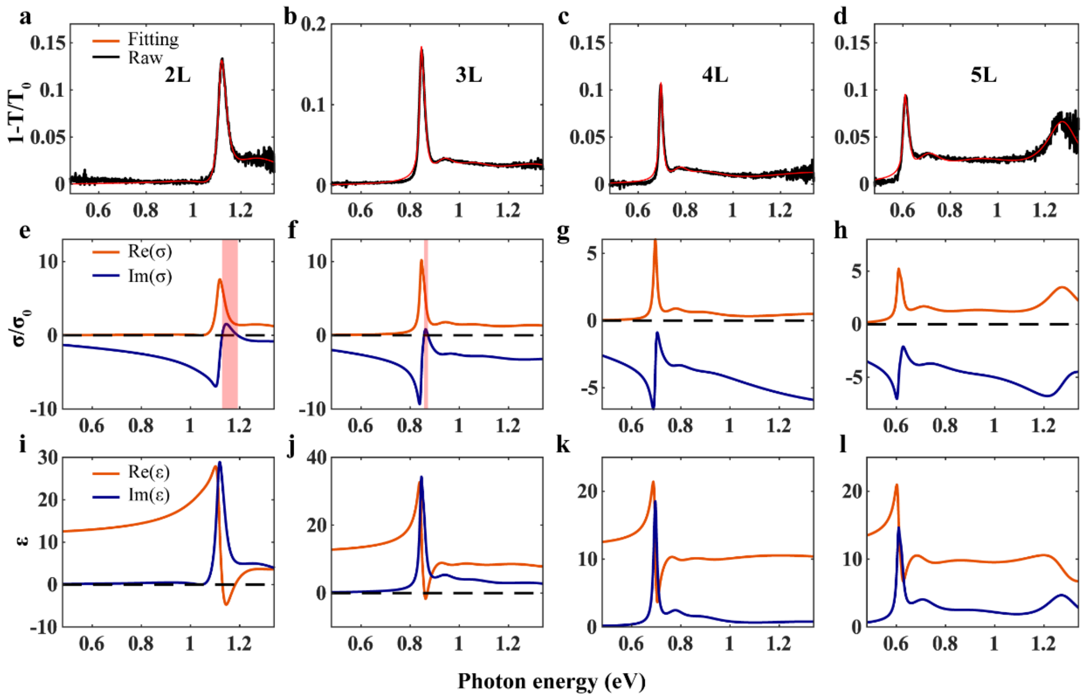

Supplementary Figure 2 | Fitting results of few-layer BP. Extinction spectra (black curves: raw data, red curves: fitting result) of BP **a** 2L, **b** 3L, **c** 4L and **d** 5L. Optical conductivities of **e** 2L, **f** 3L, **g** 4L and **h** 5L, the pink area indicate a $\mathrm{Im}(\sigma_{AC}) > 0$ regime. Dielectric functions of **i** 2L, **j** 3L **k** 4Land **l** 5L. In the fitting process, we fixed $\varepsilon(0) = 12$.

Using the same method, the optical properties for several monolayer TMDCs, including $MoSe_2$, WSe_2, WS_2 and MoS_2, are presented in Supplementary Fig. 3. To ensure further valid

comparison of oscillator strengths, the experimental data were collected under the same measurement conditions. The monolayer TMDCs samples were placed on the same enclosed Linkam chamber and the polarizer for analysis was kept at the same angle as the armchair polarization direction of the monolayer BP, even though the monolayer TMDCs are not vulnerable and insensitive to the polarization. In our analysis, the bulk interlayer spacings of $MoSe_2$ (0.646 nm), WSe_2 (0.649 nm), WS_2 (0.618 nm), and MoS_2 (0.615 nm)[8] were assumed. The fitted reflection spectra over the raw data are shown in Supplementary Figs. 3a-d, and the insets display the optical contrast values. Comparing with monolayer BP (~3%), the monolayer TMDCs (~10%) are more visible when they are placed on the same PDMS substrate. The red arrows in Figs. 3a-d indicate the $2s$ resonance states. However, the $2s$ resonance of $MoSe_2$ hides in the B exciton resonance (the second resonance peak). Corresponding fitted optical conductivities and dielectric functions are presented in Supplementary Figs. 3e-h and Supplementary Figs. 3i-l. Results agree well with earlier reports based on the reflectance measurements[8], which validates our procedure.

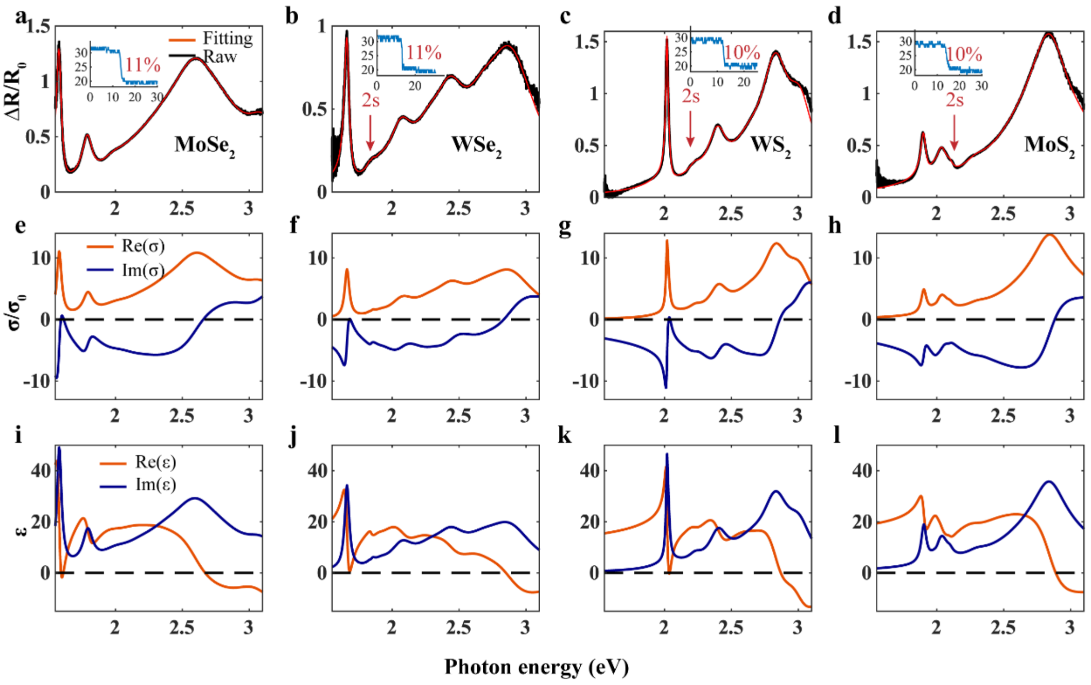

Supplementary Figure 3 | Optical properties of monolayer TMDCs. Reflection spectra (black curves: raw data, red curves: fitting result) of **a** $MoSe_2$, **b** WSe_2, **c** WS_2 and **d** MoS_2, the inset is the corresponding optical contrast. Optical conductivities of **e** $MoSe_2$, **f** WSe_2, **g** WS_2 and **h** MoS_2. Dielectric functions of **i** $MoSe_2$, with $\varepsilon(0) = 15$, **j** WSe_2, with $\varepsilon(0) = 12$, **k** WS_2, with $\varepsilon(0) = 12$ and **l** MoS_2, with $\varepsilon(0) = 15$.

The almost perfect fit has obtained by a superposition of many Lorentzian oscillators as shown in Supplementary Fig.1 to Fig.3. However, some of the oscillators don't show a clear origin. Therefore, to better gain insights on the oscillator strength and linewidth, here we reduce the number of oscillators and parameterize the dielectric function (Supplementary Equation (7)) with oscillators of clear origin, including the exciton ground state resonances, as well as the first excited state. Such practice also yields a reasonably good fitting, as shown in Supplementary Fig. 4 by the red dashed curves. This only leads to negligibly small changes in

the $\mathrm{Im}(\sigma) > 0$ energy regime, so our conclusions remain unchanged. The extracted oscillator strength (f_k), peak energy (ω_k), and linewidth (γ_k) are summarized in Supplementary Tables 1 and 2. Monolayer BP 1*s* state shows the largest oscillator strength.

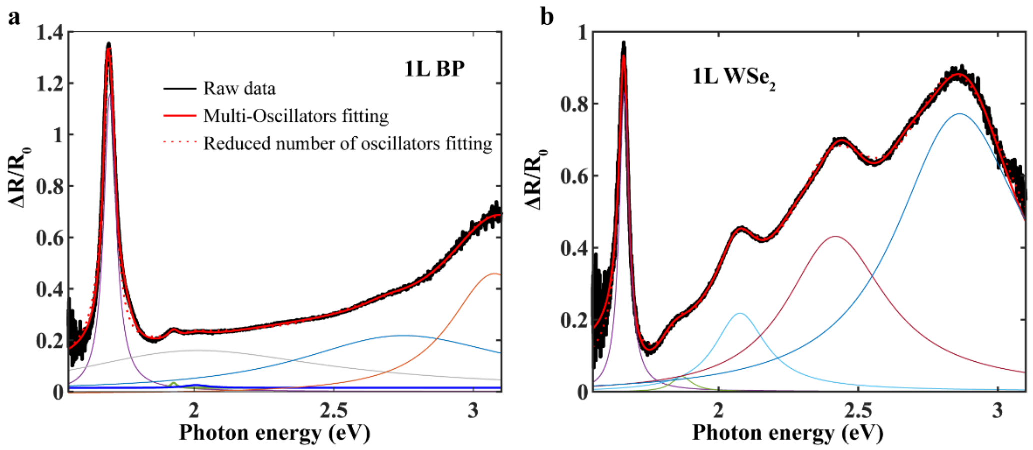

Supplementary Figure 4 | Fitting results with reduced number of oscillators. a 1L BP **b** 1L TMDCs (WSe_2). (black: raw data, red solid: fitting with a superposition of Lorentzian oscillators, red dashed: fitting results with reduced number of Lorentzian oscillators, with its individual components indicated by the color curves).

Extracted parameters of 1L BP								
1*s*			2*s*			3*s*		
f_k (eV^2)	ω_k (eV)	γ_k (eV)	f_k (eV^2)	ω_k (eV)	γ_k (eV)	f_k (eV^2)	ω_k (eV)	γ_k (eV)
4.331	1.698	0.048	0.042	1.926	0.025	0.090	2.006	0.100
Higher transition 1			Higher transition 2			Higher transition 3		
f_k (eV^2)	ω_k (eV)	γ_k (eV)	f_k (eV^2)	ω_k (eV)	γ_k (eV)	f_k (eV^2)	ω_k (eV)	γ_k (eV)
14.802	2.010	1.100	16.282	2.745	0.912	14.562	3.074	0.382
Extracted parameters of 2L BP								
1*s*			2*s*			\		
f_k (eV^2)	ω_k (eV)	γ_k (eV)	f_k (eV^2)	ω_k (eV)	γ_k (eV)	\	\	\
1.027	1.121	0.033	0.870	1.240	0.177	\	\	\
Extracted parameters of 3L BP								
1*s*			2*s*			Higher transition		
f_k (eV^2)	ω_k (eV)	γ_k (eV)	f_k (eV^2)	ω_k (eV)	γ_k (eV)	f_k (eV^2)	ω_k (eV)	γ_k (eV)
0.630	0.846	0.022	0.136	0.945	0.072	0.473	1.038	0.25
Extracted parameters of 4L BP								
1*s*			2*s*			Higher transition		
f_k (eV^2)	ω_k (eV)	γ_k (eV)	f_k (eV^2)	ω_k (eV)	γ_k (eV)	f_k (eV^2)	ω_k (eV)	γ_k (eV)

0.212	0.694	0.017	0.050	0.776	0.067	0.204	0.857	0.220
Extracted parameters of 5L BP								
$1s$			$2s$			E_{22}		
f_k (eV^2)	ω_k (eV)	γ_k (eV)	f_k (eV^2)	ω_k (eV)	γ_k (eV)	f_k (eV^2)	ω_k (eV)	γ_k (eV)
0.153	0.613	0.019	0.282	0.6900	0.159	0.873	1.276	0.160

Supplementary Table 1 | Summary of fitted oscillator strength, peak energy, and linewidth of BP. The higher transition peaks in the Supplementary Table 1 have no clear origin but just to allow the tail of spectra to be fitted. The 2*s* state of 2L BP is not obvious in our measurement due to the cut off energy of our setup. As a result, the fitting parameters of 2*s* are not so reliable.

Summary of extracted parameters						
Exciton states	$1s$			$2s$		
Parameters	f_k (eV^2)	ω_k (eV)	γ_k (eV)	f_k (eV^2)	ω_k (eV)	γ_k (eV)
1L BP	4.331	1.698	0.048	0.042	1.926	0.025
2L BP	1.027	1.121	0.033	0.870	1.240	0.177
3L BP	0.630	0.846	0.022	0.136	0.945	0.072
4L BP	0.212	0.694	0.017	0.050	0.776	0.067
5L BP	0.153	0.613	0.019	0.282	0.6900	0.168
1L $MoSe_2$	2.939	1.579	0.041	\	\	\
1L WSe_2	2.163	1.662	0.040	0.260	1.868	0.088
1L WS_2	2.059	2.025	0.024	0.163	2.200	0.062
1L MoS_2	1.292	1.902	0.047	0.197	2.097	0.055

Supplementary Table 2 | Comparison of 1s and 2s states among BP and 1L TMDCs. The 2*s* state of 1L $MoSe_2$ is not available in our measurement.

Supplementary Note II: Extraction of the exciton binding energy from the 1*s*-2*s* energy difference

Within the Wannier-Mott framework, excitons in atomically thin BP obey the Schrödinger equation[3, 9]:

$$\left[-\frac{1}{\mu_x}\frac{\partial^2}{\partial x^2}-\frac{1}{\mu_y}\frac{\partial^2}{\partial y^2}+V_{eh}(r)\right]\Psi_m(x,y)=E_m\Psi_m(x,y), \qquad (8)$$

Where $\mu_{x(y)}=\left(1/m_{x(y)}^e+1/m_{x(y)}^h\right)^{-1}$ is the reduced exciton mass of *M*-layer BP in the armchair and zigzag directions, with conduction and valance band effective masses given by $m_{x(y)}^e$ and $m_{x(y)}^h$. For atomically thin BP on the PDMS substrate, considering the nonlocal screening effect[10-12], the electron-hole interaction potential takes a form of

$$V_{eh}(r)=\frac{2\pi}{(\varepsilon_{\mathrm{top}}+\varepsilon_{\mathrm{sub}})r_0}\left[H_0\left(\frac{r}{r_0}\right)-Y_0\left(\frac{r}{r_0}\right)\right], \qquad (9)$$

where $r=(x^2+y^2)^{\frac{1}{2}}$ is the electron-hole separation, H_0 and Y_0 are Struve and Neumann functions respectively, $r_0=d\varepsilon_{\mathrm{BP}}/(\varepsilon_{\mathrm{top}}+\varepsilon_{\mathrm{sub}})$ is the screening length and d is the thickness of BP film, $\varepsilon_{\mathrm{top}}=\varepsilon_0$ for top vacuum environment and $\varepsilon_{\mathrm{sub}}=1.93\varepsilon_0$ for the PDMS substrate. In our theoretical model, the parameter $\beta=d_1\varepsilon_{\mathrm{BP}}$ is adjusted to fit the experimental data, where d_1 is the single layer thickness of BP (for *M*-layer, thickness: $d=Md_1$). β is supposed to be a multiplication between the thickness of each layer and the dielectric constant of BP. Based on our previous study on 2L-6L BP's exciton binding energy[12], $\beta=52\varepsilon_0$ Å (corresponding to 5.2 Å for each layer thickness and $10\varepsilon_0$ for BP dielectric constant) give the best global fitting of 2L-6L BP, and the exciton binding energy in freestanding monolayer BP ($\varepsilon_{\mathrm{sub}}=\varepsilon_0$) is predicted to be ~762 meV. Now since we have monolayer data, we can adjust our model to globally fit 1L-6L BP, then $\beta=78\varepsilon_0$ Å gives a better global fitting result. This yields

an exciton binding energy of ~452 meV and Δ_{12} of ~219 meV for monolayer BP on PDMS. In this case ($\beta = 78\varepsilon_0$ Å), the freestanding monolayer BP has a binding energy of ~560 meV and Δ_{12} of ~239 meV. The fitting parameter $\beta = 78\varepsilon_0$ Å brings the energy closer to the DFT calculation results[13] as well, thus the corresponding binding energy value is more reliable than just simply extrapolating the 2L-6L results obtained previously to monolayer. The global fitting is very good, as shown in Supplemental Fig. 5, where the energy difference Δ_{12} of 1L-6L BP are plotted for both calculated and experimental values. The calculated Δ_{12} (with $\beta = 78\varepsilon_0$ Å) agrees very well with experimental observations. 1*s* exciton binding energies are shown as well.

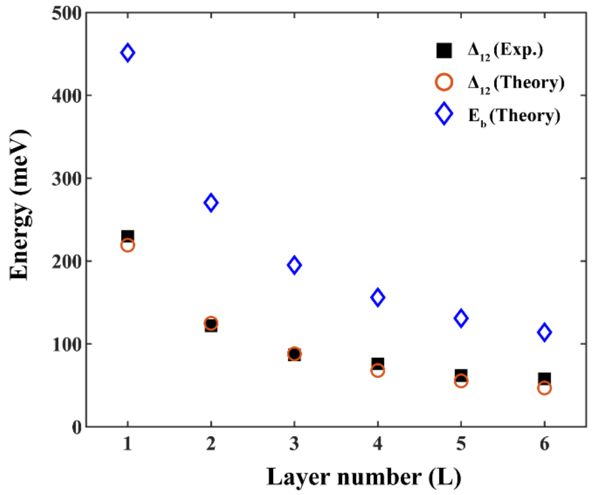

Supplementary Figure 5 | Extraction of the exciton binding energy. Experimentally and theoretically obtained 1*s*-2*s* energy difference Δ_{12}, and exciton binding energy as a function of layer number (L).

Supplementary Note III. Derivation of the HEPs dispersion in anisotropic BP

In this note, we derive the dispersion relation for the EPs in anisotropic BP, which is placed between vacuum ($\varepsilon_{\text{vac}} = 1$) and a PDMS substrate ($\varepsilon_{\text{sub}} = 1.93$). We also determine the propagating direction of HEPs from the hyperbola asymptote angles.

We utilize the loss function to determine the polaritons dispersion. The loss function $-\text{Im}(1/\varepsilon)$, is related to the imaginary part of the inverse of the random phase approximation (RPA) dielectric function[14-17]. The **q**-dependent dielectric function has the following form in 2D case for the high symmetry directions:

$$\varepsilon(\mathbf{q}, \omega) = \varepsilon_{\text{env}} + \frac{i\sigma(\omega)}{\varepsilon_0 \omega}\frac{\mathbf{q}}{2}, \tag{10}$$

where ε_{env} is the permittivity of the environment ($\varepsilon_{\text{env}} = (1 + \varepsilon_{\text{sub}})/2$), ε_0 is the vacuum permittivity, and $\mathbf{q}$ is the wave vector. The sheet optical conductivity $\sigma(\omega)$ is extracted from the reflection contrast spectrum as mentioned in Supplementary Note I. The calculated loss functions along the two principal axes are plotted as a pseudo-color map in Fig. 3a in the main text. We trace the maxima in the map for armchair direction with a curve, which is the polariton dispersion curve $\omega(\mathbf{q})$ in the armchair direction, as represented by the black dashed curve in Fig. 3a in the main text. There is no clear maximum for the zigzag direction loss function map, which suggests no polariton mode exists in that direction.

To quantify the basic properties of exciton-polaritons, the quality factor $Q = \frac{\omega}{\Delta\omega}$ were calculated[18, 19], where ω and $\Delta\omega$ are the resonance energy and linewidth of the exciton-polaritons. We plot the loss function values versus frequency at a fixed wave vector in

Supplementary Fig. 6. The Q values were then extracted from the peak energy ω and linewidth $\Delta\omega$ of each curve. At a near zero wave vector, the Q factor can reach as high as 35.52, which is reasonably good. As the wave vector increases, the Q factor decreases.

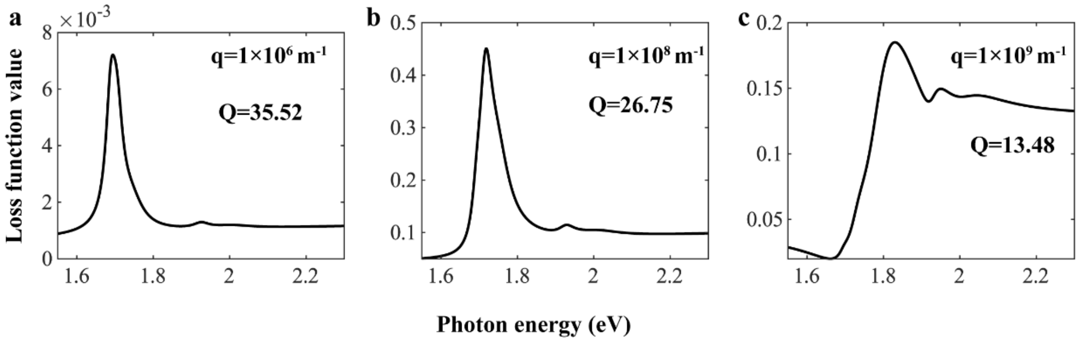

Supplementary Figure 6 | Quality factor of exciton-polaritons in monolayer BP. The exciton-polariton loss function at **a** $\mathbf{q} = 1 \times 10^6\ m^{-1}$, **b** $\mathbf{q} = 1 \times 10^8\ m^{-1}$, **c** $\mathbf{q} = 1 \times 10^9\ m^{-1}$. The Q value is determined by $\mathrm{Q} = \frac{\omega}{\Delta\omega}$ and the Q value becomes smaller at larger wave vectors.

Next, let us consider the characteristics of HEPs that can be supported by a hyperbolic material. The low symmetry of BP allows for two independent in-plane components of the optical conductivities, $\sigma_{\mathrm{AC}}(\omega)$ and $\sigma_{\mathrm{ZZ}}(\omega)$. We assume that the wave vector of HEP is at an angle θ with respect to the armchair axis, in which case the wave vector $\mathbf{q}$ is no longer along the principal axes, but along a certain direction. With the magnitude of $q = \sqrt{q_{\mathrm{AC}}^2 + q_{\mathrm{ZZ}}^2}$, $q_{AC} = q\cos\theta$, $q_{\mathrm{ZZ}} = q\sin\theta$, and the sheet optical conductivity can be rewritten as:

$$\sigma = \sigma_{\mathrm{AC}}\cos^2\theta + \sigma_{\mathrm{ZZ}}\sin^2\theta, \tag{11}$$

then $\varepsilon(\mathbf{q}, \omega)$ reads:

$$\varepsilon(\mathbf{q}, \omega) = \varepsilon_{\mathrm{env}} + \frac{i(\sigma_{\mathrm{AC}}\cos^2\theta + \sigma_{\mathrm{ZZ}}\sin^2\theta)}{\varepsilon_0\omega}\frac{\mathbf{q}}{2}, \tag{12}$$

At each frequency ω, $\sigma_{\mathrm{AC}}(\omega)$ and $\sigma_{\mathrm{ZZ}}(\omega)$ values were obtained from the reflection contrast spectra under the armchair and zigzag polarization, the loss function is then calculated by

substituting Supplementary Equation (12) into $-\mathrm{Im}(1/\varepsilon)$. The calculated loss functions at three representative frequencies ($E = 1.71$ eV, $E = 1.75$ eV, $E = 1.79$ eV) are displayed as pseudo-color maps in Supplementary Figs. 7a-c, the blue solid curves represent the maxima in the loss values. Then, the maximal values in the map are extracted as the iso-frequency contour (curves of Fig. 3b in the main text) in **q**-space. After establishing the hyperbolic iso-frequency contours, the corresponding angles θ were extracted from the hyperbola asymptotes, and the direction of HEP beams are normal to the hyperbola asymptotes, with angles $\frac{\pi}{2} - \theta$ or $\frac{\pi}{2} + \theta$ (see arrows in Fig. 3b in the main text). In our case, both the real and imaginary parts of optical conductivity are included, so the angle θ deviates the common definition $\theta = arctan\frac{\mathrm{Im}(\sigma_{AC})}{\mathrm{Im}(\sigma_{ZZ})}$, as shown in Supplementary Fig. 7d.

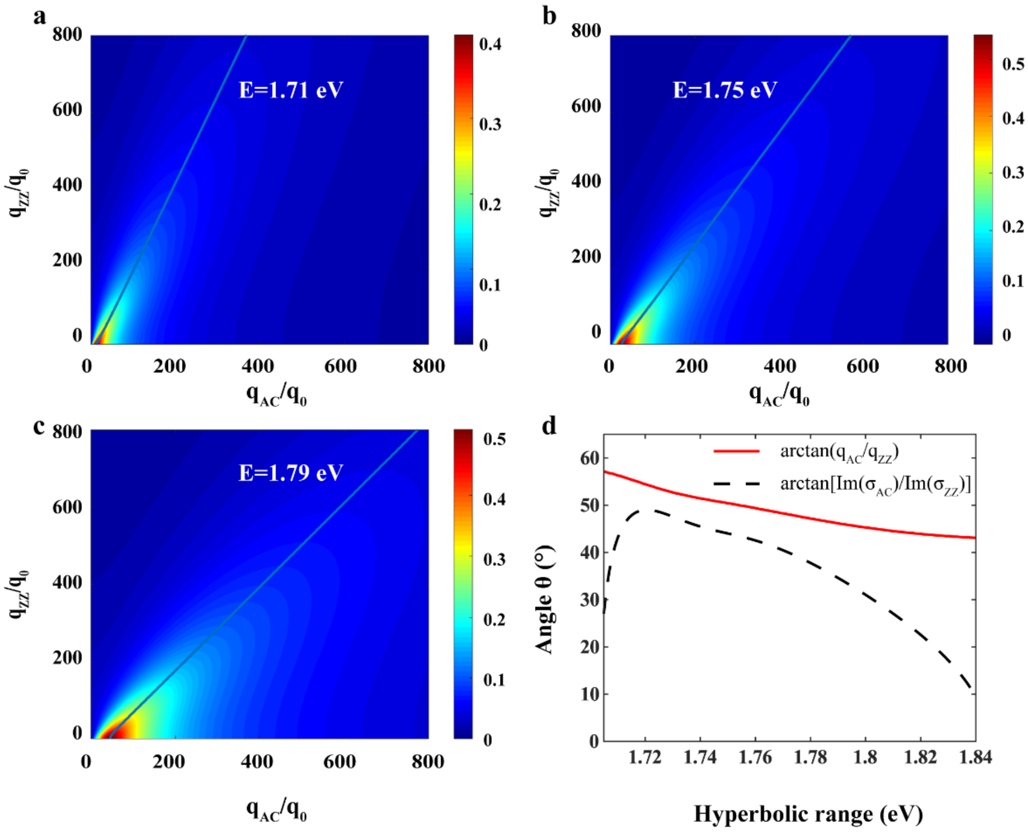

Supplementary Figure 7 | Characteristic of HEPs at fixed energy. The calculated loss function is displayed as a pseudo-color map at **a** $E = 1.71$ eV, **b** $E = 1.75$ eV, **c** $E = 1.79$ eV. The blue solid

curves trace the maximal values of the loss functions. **d** The angles θ in the hyperbolic regime. The red solid curve is obtained from the asymptotes of the iso-frequency curves, where both real and imaginary parts of the optical conductivity are included; the black dashed curve only reflects the imaginary parts of the optical conductivity, where $\theta = arctan\frac{\mathrm{Im}(\sigma_{\mathrm{AC}})}{\mathrm{Im}(\sigma_{\mathrm{ZZ}})}$.

Supplementary Note Ⅳ. Exciton absorption in monolayer BP

For few-layer BP, the frequency-integrated real part of the optical conductivity (exciton absorption) is proportional to the exciton binding energy, which provides an additional scheme to estimate the exciton binding energy. Consistent with the method in reference[20], the real part of the optical conductivity from 1L-3L BP are shown in Supplementary Fig. 8 and the integrated area values are labeled. The frequency-integrated conductivity is consistent with the trend of other few-layer BP samples, which further validates that it is indeed a monolayer BP with high quality and the corresponding exciton binding energy obtained by us is reliable.

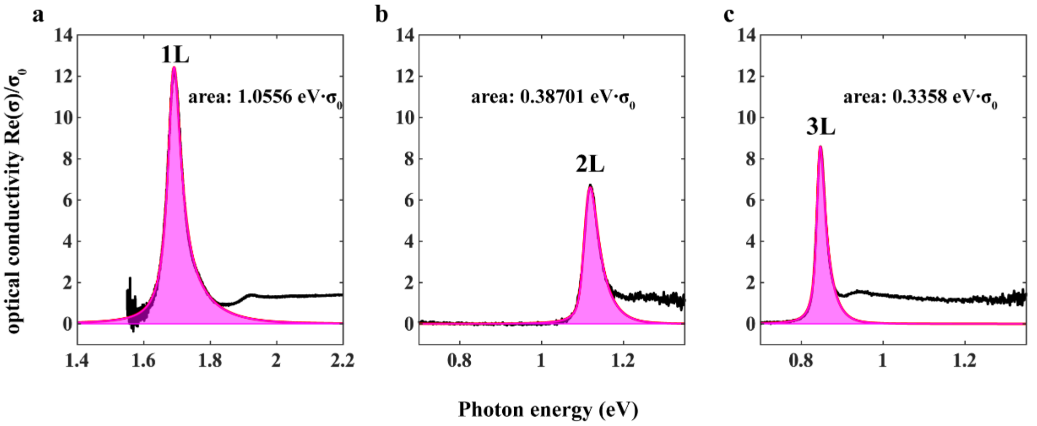

Supplementary Figure 8 | Exciton absorption in monolayer and few-layer BP. The frequency-integrated real part of the optical conductivity (Re(σ)) of the 1s exciton for **a** 1L, **b** 2L, **c** 3L BP, in the unit of $\sigma_0 = e^2/4\hbar$. The exciton oscillator strength is proportional to the integrated area of the 1s exciton peak, as indicated by the shaded areas.

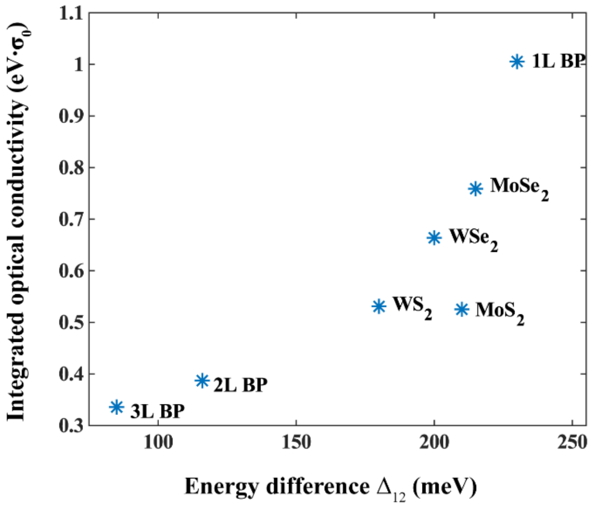

Supplementary Figure 9 | Comparison of the 1*s*-2*s* energy difference (an indication of exciton binding energy) and the integrated optical conductivities (exciton absorption) among TMDCs and BP. The energy difference Δ_{12} are extracted from the experimental resonance peaks directly. The experimental Δ_{12} of $MoSe_2$ is not available in our spectra, and the data is taken from the reference[21]. 1L BP has the strongest exciton resonance features.

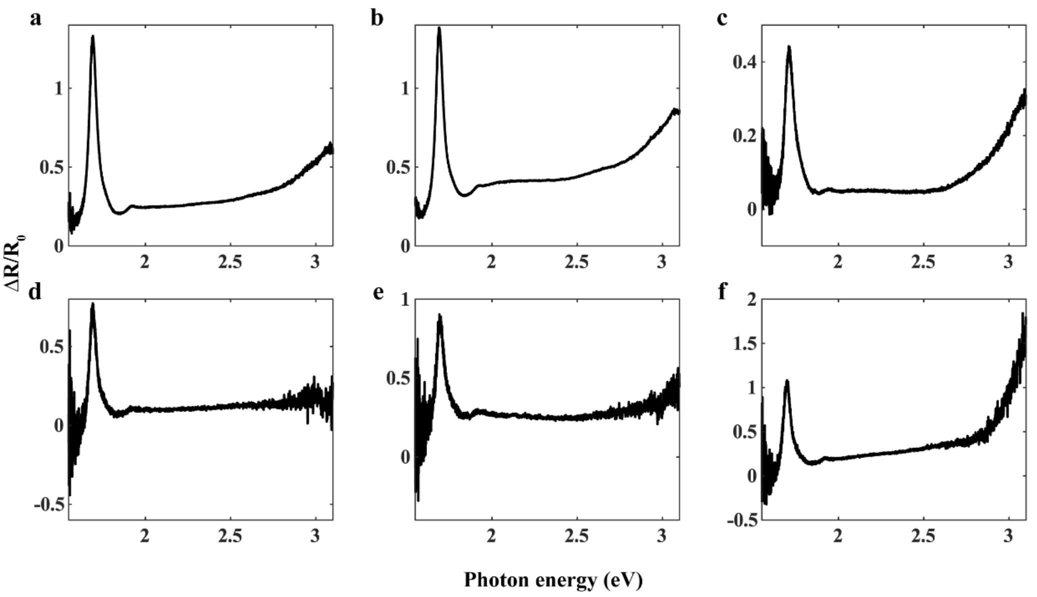

Supplementary Figure 10 | Additional spectra for monolayer BP. The smaller intensities in Supplementary Figs. 10c-f are attributed to the smaller sample size, where the sample area is not large enough to fill the aperture size. Consequently, the resulting reflected light from the sample is less. However, the exciton resonance energies of 1*s* and 2*s* states are not affected.

Supplementary References:

1. Li Y, Heinz TF. Two-dimensional models for the optical response of thin films. *2D Mater.* **5**, 025021 (2018).

2. Deilmann T, Thygesen KS. Unraveling the not-so-large trion binding energy in monolayer black phosphorus. *2D Mater.* **5**, 041007 (2018).

3. Chaves A, Low T, Avouris P, Çakır D, Peeters FM. Anisotropic exciton Stark shift in black phosphorus. *Phys. Rev. B* **91**, 155311 (2015).

4. Asahina H, Morita A. Band structure and optical properties of black phosphorus. *J. Phys. C: Solid State Phys.* **17**, 1839 (1984).

5. Kumar P, Bhadoria BS, Kumar S, Bhowmick S, Chauhan YS, Agarwal A. Thickness and electric-field-dependent polarizability and dielectric constant in phosphorene. *Phys. Rev. B* **93**, 195428 (2016).

6. T. Nagahama, M. Kobayashi, Y. Akahama, S. Endo, Narita S. Optical determination of dielectric constant in black phosphorus. *J. Phys. Soc. Jpn.* **54**, 2096 (1985).

7. Castellanos-Gomez A*, et al.* Isolation and characterization of few-layer black phosphorus. *2D Mater.* **1**, 025001 (2014).

8. Li Y*, et al.* Measurement of the optical dielectric function of monolayer transition-metal dichalcogenides:MoS2,MoSe2,WS2, andWSe2. *Phys. Rev. B* **90**, 205422 (2014).

9. Li P, Appelbaum I. Electrons and holes in phosphorene. *Phys. Rev. B* **90**, 115439 (2014).

10. He K*, et al.* Tightly bound excitons in monolayer WSe(2). *Phys. Rev. Lett.* **113**, 026803 (2014).

11. Chernikov A*, et al.* Exciton binding energy and nonhydrogenic Rydberg series in monolayer WS(2). *Phys. Rev. Lett.* **113**, 076802 (2014).

12. Zhang G, Huang S, Wang F, Xing Q, Low T, Yan H. Determination of layer-dependent exciton binding energies in few-layer black phosphorus. *Sci. Adv.* **4**, eaap9977 (2018).

13. Qiu DY, da Jornada FH, Louie SG. Environmental Screening Effects in 2D Materials: Renormalization of the Bandgap, Electronic Structure, and Optical Spectra of Few-Layer Black Phosphorus. *Nano Lett.* **17**, 4706-4712 (2017).

14. Wang C*, et al.* Van der Waals thin films of WTe2 for natural hyperbolic plasmonic surfaces. *Nat. Commun.* **11**, 1158 (2020).

15. Ma W*, et al.* In-plane anisotropic and ultra-low-loss polaritons in a natural van der Waals crystal. *Nature* **562**, 557-562 (2018).

16. Hu G*, et al.* Topological polaritons and photonic magic angles in twisted alpha-MoO3 bilayers. *Nature* **582**, 209-213 (2020).

17. Berkowitz ME*, et al.* Hyperbolic Cooper-Pair Polaritons in Planar Graphene/Cuprate Plasmonic Cavities. *Nano Lett.* **21**, 308-316 (2021).

18. Low T*, et al.* Polaritons in layered two-dimensional materials. *Nat. Mater.* **16**, 182-194 (2017).

19. Basov DN, Fogler MM, Garcia de Abajo FJ. Polaritons in van der Waals materials. *Science* **354**, 195 (2016).

20. Zhang G*, et al.* The optical conductivity of few-layer black phosphorus by infrared spectroscopy. *Nat. Commun.* **11**, 1847 (2020).

21. Arora A, Nogajewski K, Molas M, Koperski M, Potemski M. Exciton band structure in layered MoSe2: from a monolayer to the bulk limit. *Nanoscale* **7**, 20769-20775 (2015).
